# Genetic diversity and phylogeography of Siberian roe deer, *Caproulus pygargus*, in central and peripheral populations

**DOI:** 10.1002/ece3.2458

**Published:** 2016-09-22

**Authors:** Yun Sun Lee, Nickolay Markov, Alexander Argunov, Inna Voloshina, Damdingiin Bayarlkhagva, Baek‐Jun Kim, Mi‐Sook Min, Hang Lee, Kyung Seok Kim

**Affiliations:** ^1^ Conservation Genome Resource Bank for Korean Wildlife College of Veterinary Medicine and Research Institute for Veterinary Science Seoul National University Seoul Korea; ^2^ Institute of Plant and Animal Ecology Urals Branch of Russian Academy of Sciences Yekaterinburg Russia; ^3^ Institute for Biological Problems of Cryolihtozone Siberian Branch of Russian Academy of Sciences Yakutsk Russia; ^4^ Lazovsky State Nature Reserve Lazo Primorsky Krai Russia; ^5^ Department of Molecular Biology and Genetics National University of Mongolia Ulaanbaatar Mongolia; ^6^ Department of Climate and Ecology National Institute of Ecology Maseo‐myeon Seocheon‐gun Chungcheongnam‐do Korea; ^7^ Department of Ecology, Evolution, and Organismal Biology Iowa State University Ames IA USA

**Keywords:** *Capreolus pygargus*, genetic diversity, mitochondrial DNA, Siberian roe deer

## Abstract

Current understanding of phylogeographical structure and genetic diversity of Siberian roe deer remains limited mainly due to small sample size and/or low geographical coverage in previous studies. Published data suggest at least two phylogroups: western (Ural Mountains and Western Siberia) and eastern (east from lake Baikal, including the Korean peninsula), but their phylogenetic relationship remains unclear. Combined sequences of cytochrome *b* (1140 bp) and the mtDNA control region (963 bp) were analyzed from 219 Siberian roe deer from 12 locations in Russia, Mongolia, and South Korea, which cover a large part of its range, to assess genetic diversity and phylogeographical status. Special emphasis was placed on the demographic history and genetic features of central, peripheral, and isolated populations. Results of median‐joining network and phylogenetic tree analyses indicate that Siberian roe deer from the Urals to the Pacific Ocean are genetically diverse and that geographical distribution and composition of haplogroups coincide with previously described ranges of the subspecies *Capreolus pygargus pygargus* and *Capreolus pygargus tianschanicus*. We found that peripheral populations in the northwestern parts of the species range (Urals), as well as the isolated population from Jeju Island, are genetically distinct from those in the core part of the range, both in terms of genetic diversity and quantitative composition of haplogroups. We also found that northwestern (Urals) and northern (Yakutia) peripheral populations share the same haplogroup and fall into the same phylogenetic clade with the isolated population from Jeju Island. This finding sheds light on the taxonomic status of the Jeju Island population and leads to hypotheses about the discordance of morphological and genetic evolution in isolated populations and specific genetic features of peripheral populations.

## 1 Introduction

**Figure 1:**
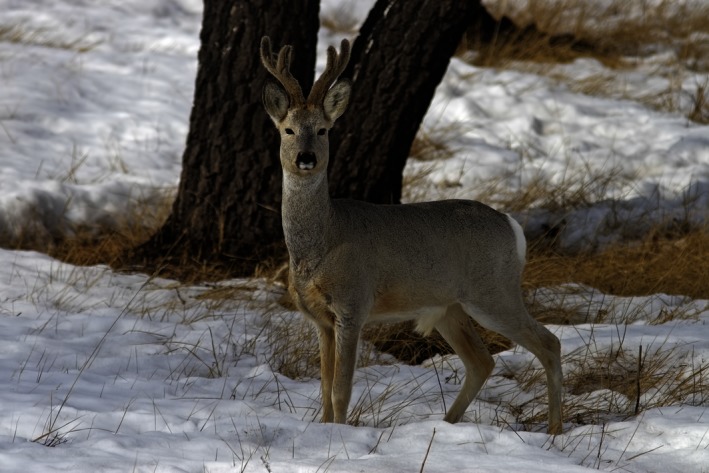
A male Siberian roe deer (*Caproulus pygargus*) in Urals, Russia (Sverdlovkaya oblast’). Source: Alexey Gurinin

The roe deer (*Capreolu*s, Gray 1821) is one of the most widespread artiodactyl genera. It includes two species: the European roe deer (*Capreolu*s *capreolus*) and the Siberian roe deer (*Capreolu*s *pygargus*; Fig. 1). The Siberian roe deer is widely distributed in continental Asia and parts of Eastern Europe, from the Khoper River and Don River bend to the Ural Mountains and across southern Siberia. It is found through northern Mongolia and east to the coastlines of the East Sea, and the Yellow Sea, including the Korean Peninsula (Danilkin, 1999). It ranges geographically from the West Siberian Plain south to Lake Balkhash, and east from there well into Kazakhstan without reaching the Aral Sea. It also ranges from Manchuria through Northern and Central China to the western half of the left bank of the Yangtze River and into eastern Tibet (Bannikov, 1954; Sokolov, Danilkin, & Dulamtseren, 1982; Dulamtseren, Tsendjav, & Avirmed, 1989 [cit. by Danilkin, 1999]). Records from further south as far as northeastern Myanmar require confirmation. It also occurs on Jeju Island in South Korea.

Although intraspecific taxonomy of the Siberian roe deer is debated, most authors agree that *C. pygargus* consists of at least two subspecies with a different number of B‐chromosomes (Groves & Grubb, 2011): *C. p. pygargus*, distributed from the Volga River to lake Baikal; and *C. p. tianschanicus* found in Tien Shan, Mongolia, Trans‐Baikalia, Russian Far East, and China (Danilkin, 1999; Sheremetyeva & Sheremetyev, 2008). Roe deer in Central China and Tibet are sometimes described as a separate subspecies, *C. p. melanotis* (Danilkin, 1999; Geptner, Nasimovich, & Bannikov, 1961; Sheremetyeva, Sheremetyev, Kartavtseva, & Zhuravlev, 2010); however, its status and range boundaries are controversial and unclear (Danilkin & Markov, 1987; Sokolov & Gromov, 1990; Sokolov et al., 1986). A fourth subspecies designation, *C. p. ochracea*, has been suggested for the isolated population of roe deer on Jeju Island, South Korea (Koh & Randi, 2001).

Data on the genetics of Siberian roe deer are scarce compared to the European roe deer. Randi, Pierpaoli, and Danilkin (1998) concluded that Siberian roe deer can be divided into two major clusters, namely the eastern cluster from the Amur region of the Russian Far East and the western cluster from the Kurgan region of Western Siberia. Molecular genetics studies on the taxonomic status of the Siberian roe deer from Jeju, South Korea, and the genetic structure of populations from Northern Eurasia have been reported (Koh & Randi, 2001; Koh et al., 2013; Tokarskaia et al., 2000). Based on RAPD markers, Petrosian, Tokarskaia, Danilkin, and Ryskov (2002) confirmed the appropriateness of assigning the western and eastern groups into the subspecies *C. p. pygargus* and *C. p. tianschanicus,* respectively. On the other hand, Xiao, Zhang, Fu, and Koh (2007) argued that the Siberian roe deer found in Northeastern China belongs to the subspecies *C. p. manchuricus*, based on morphological differences from the other subspecies. Sheremetyeva et al. (2010) presented a complex phylogenetic structure of roe deer populations in the Russian Far East based on genetic analysis of the short fragment of the mtDNA control region.

Based on combined alignment of the control region and cytochrome *b*, Zvychainaya, Danilkin, Kholodova, Sipko, and Berber (2011) found three haplogroups among 79 Siberian roe deer sampled from 23 regions of Asia, including Russia and Kazakhstan. Individuals from the Russian Far East, northeastern Russia, and Trans‐Baikalia formed a single haplogroup, whereas specimens from the Urals, and Western and Central Siberia shared two distinct haplogroups. Lorenzini, Garofalo, Qin, Voloshina, and Lovari (2014) suggested three haplogroups for Siberian roe deer are distributed throughout the entire range of this species, including Western Russia, Kyrgyzstan, Northeastern China, Central‐eastern China, and Eastern Russia, but no geographical structuring of the species lineages was found.

Most of the above mentioned studies are based on relatively small sample sizes (but see Xiao et al., 2007), and this could be one reason for uncertainty in the possible phylogeographical patterns reported (particularly by Zvychainaya et al., 2011). Together, published data suggest the existence of at least two or three phylogroups, but their phylogenetic relationships remain unclear, particularly in Central Siberia where the geographical ranges of *C. p. pygargus* and *C. p. tianschanicus* may overlap (Sheremetyeva et al., 2010).

As for peripheral populations of Siberian roe deer, the picture becomes especially complex. For example, Zvychainaya et al. (2011) reported that roe deer from Urals and Trans‐Urals region (Sverdlovsk and Kurgan regions, close to the western periphery of the species’ geographical range) are represented by two haplogroups, each occupying a distal position on the phylogenetic tree. Likewise, recent data on the genetic features of roe deer from Yakutia, at the northern periphery of the species’ geographical range, put them into the Far Eastern clade (Zvychainaya et al., 2011). Thus, the phylogeographical structure of the Siberian roe deer remains ambiguous and many authors emphasize the need for extensive studies of the species in a number of regions.

In this study, we report original data from the Urals, Western and Central Siberia, the Russian Far East and Korea, including both mainland Korea and the isolated population on Jeju Island, covering most part of the geographical range of Siberian roe deer. We used a sufficient number of samples (not less than 20 from most regions) to provide reliable estimates of genetic diversity and analyzed phylogeographical patterns of the Siberian roe deer across Northern Asia, with special interest in genetic differentiation among central, peripheral, and isolated populations. In particular, we tested the hypothesis suggested by Lorenzini et al. (2014) that there is a lack of geographical structuring of genetic lineages in Siberian roe deer. We also address the question of taxonomic status of the Siberian roe deer on Jeju Island, based on data collected from across the species’ entire geographical range.

## 2 Materials and Methods

### 2.1 Sample collection and DNA analysis

**Figure 2:**
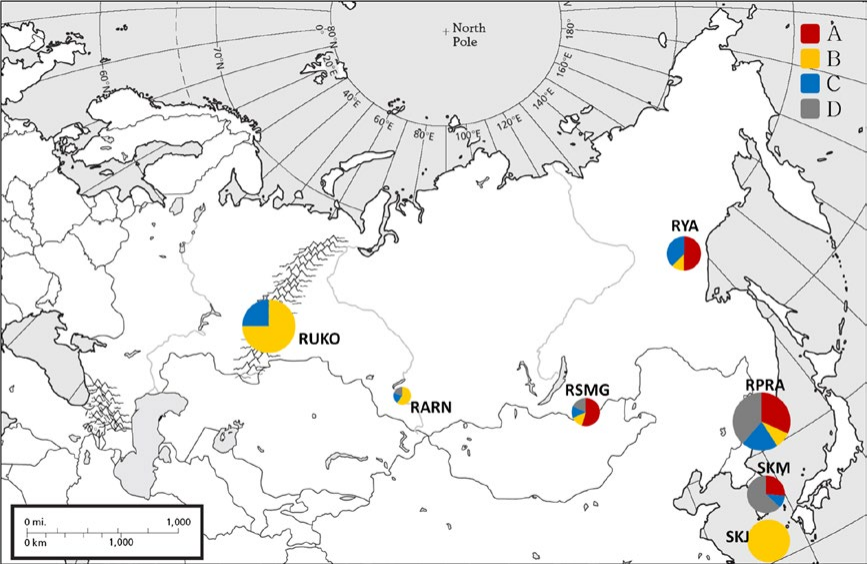
Geographical distribution of the haplogroups revealed by Bayesian analysis. The proportion of color in each circle indicates the proportion of haplogroups from the respective phylogenetic clusters (A, B, C) and all the rest (D). Circle size is proportional to the number of samples from each region. See Table 1 for regional abbreviations

We obtained tissue, blood and skin samples from 219 individuals of *C. pygargus* from 12 locations in Russia, Mongolia, and South Korea (Fig. 2, Table 1). These locations were grouped into seven regions according to geographical proximity: Jeju Island in South Korea (SKJ), South Korea mainland (SKM), Primorsky Krai and Amur region of Russia (RPRA), Yakutia in Russia (RYA), the Sokhondinsky Nature Reserve in the Trans‐Baikal region of Russia and Northern Mongolia (RSMG), Altay and Novosibirsk in Russia (RARN), and the Urals, Kurgan, and Orenburg in Russia (RUKO). All samples were stored in a −70°C freezer at the Conservation Genome Resource Bank for Korean wildlife until DNA extraction.

**Table 1:** Siberian roe deer sample information and haplotype distribution for each location per region

Region	Location (Abbreviation)	*N*	Haplotype
SKJ	South Korea, Jeju (SKJ)	37	Hap80(15), Hap81(1), Hap82(6), Hap83(2), Hap84(3), Hap85(4), Hap86(1) Hap87(4), Hap88(1)
SKM	South Korea, mainland (SKM)	30	Hap17(1), Hap19(3), Hap20(1), Hap33(1), Hap34(1), Hap45(1), Hap49(3), Hap50(1), Hap51(4), Hap52(1), Hap53(3), Hap54(1), Hap55(1), Hap56(1), Hap58(3), Hap59(1), Hap60(1), Hap95(1), Hap108(1)
RPRA	Russia, PrimorskyKrai (RPR)	41	Hap1(1), Hap5(1), Hap6(1), Hap7(1), Hap18(1), Hap21(1), Hap24(1), Hap25(1),Hap26(1), Hap28(1), Hap36(1), Hap37(1), Hap38(1), Hap39(1), Hap42(3), Hap43(1), Hap46(3), Hap48(1), Hap57(1), Hap61(1), Hap62(2), Hap63(1), Hap65(2), Hap66(1), Hap67(1), Hap68(1), Hap69(1), Hap70(1), Hap71(1), Hap90(1), Hap92(1), Hap93(1), Hap94(1), Hap96(1), Hap97(1)
RPRA	Russia, Amur region (RAM)	10	Hap3(1), Hap9(1), Hap22(1), Hap31(1), Hap44(1), Hap47(2), Hap91(1), Hap104(1), Hap105(1)
RYA	Russia, Yakutia (RYA)	24	Hap10(7), Hap11(1), Hap29(3), Hap30(1), Hap89(5), Hap101(5), Hap102(1), Hap107(1)
RSMG	Russia, Sokhondinsky (RSO)	10	Hap12(1), Hap13(1), Hap23(1), Hap40(1), Hap41(1), Hap64(1), Hap74(1), **Hap98**(1), Hap103(1), Hap106(1), Hap109(1), Hap110(1)
RSMG	Mongolia, Northern part (MGN)	12	Hap2(1), Hap4(1), Hap8(1), Hap14(1), Hap15(1), Hap16(1), Hap27(1), Hap32(1), Hap35(1), Hap72(1)
RARN	Russia, Altay (RAL)	3	**Hap75**(1), **Hap98**(1), Hap112(1)
RARN	Russia, Novosibirsk (RNO)	6	**Hap75**(4), **Hap79**(1), Hap111(1)
RUKO	Russia, Ural (RUR)	23	**Hap73**(2), **Hap75**(5), **Hap76**(1), **Hap79**(3), **Hap99**(4), **Hap100**(8)
RUKO	Russia, Kurgan (RKU)	20	**Hap73**(6), **Hap75**(1), **Hap77**(1), Hap78(1), **Hap79**(2), **Hap99**(8), **Hap100**(1)
RUKO	Russia, Orenburg (ROR)	3	**Hap75**(1), **Hap76**(1), **Hap77**(1)
C.c	Ukraine, Crimea	3	Hap113(2), Hap114(1)

Mitochondrial DNA control region and cytochrome *b* were combined for analysis. Bold indicate haplotypes shared among regions.

*N*, sample size; *C. c*,* Capreolus capleolus* (out‐group).

Genomic DNA was extracted from samples using the QIAamp tissue kit (Qiagen, Germany). The mitochondrial cytochrome *b* gene (1,140 bp) was amplified by polymerase chain reaction (PCR) using universal primers L14724 (5′—GAT ATG AAA AAC CAT CGT TG—3′) and H15915 (5′—AAC TGC AGT CAT CTC CGG TTT ACA AGA C—3′) (Kocher et al.,^1989^). PCR conditions were as follows: 94°C for 4 min; 35 cycles of 94°C for 30 s, 55°C for 30 s, and 72°C for 1 min; and finally 72°C for 5 min. A 923‐bp fragment of the mtDNA control region was amplified using primers L15775 (5′—ACA TGA ATT GGA GGA CAA CCA GT—3′) (Irwin, Kocher, & Wilson, 1991) and H651 (5′—AAG GCT AGG ACC AAA CCT—3′) (Kocher et al., 1989). PCR conditions were as follows: 94°C for 5 min; 35 cycles of 94°C for 45 s, 55°C for 45 s, and 72°C for 1 min 30 s; and finally 72°C for 5 min. Amplification was carried out in 20 μl reaction volume containing 10–100 ng template DNA, 100 μmol/L each dNTPs, 10 pmole of each primer, 1.5 mmol/L MgCl_2_, 1 unit i‐star Taq™ DNA polymerase (iNtRON Biotechnology Inc, Korea), and 1 ×  PCR buffer. PCR products were purified using the Zymoclean™ Gel DNA Recovery Kit (ZYMO RESEARCH, USA). Purified PCR products were sequenced using an ABI Prism™ 377 automated sequencer (Applied Biosystems Inc, USA). The sequencing primers for both mtDNA regions were the same as those used for the amplification, except that in the case of the control region, the sequencing primers included a supplementary inner primer: L—362 (5′—AAT CAC CAT GCC GCG TGA AAC C—3′) (Douzery & Randi, 1997).

### 2.2 Data analysis

The sequences derived in this study were identified as being from *Capreolus* species through BLAST searches (Altschul et al., 1997). Sequences were aligned with Clustal X version 1.83 (Thompson, Gibson, Plewniak, Jeanmougin, & Higgins, 1997). All downstream analyses were conducted with concatenated sequences of the two mtDNA regions (2063 bp).

Haplotype diversity (*h*) and nucleotide diversity (π) for each of geographical samples were estimated with DNASP version 5.1 (Librado & Rozas, 2009). ARLEQUIN 3.1 (Excoffier, Laval, & Schneider, 2005) was used to calculate mismatch distributions and pairwise *F*_ST_s to compare genetic differentiation among geographical regions. Mismatch distributions were analyzed using the sudden expansion model (Rogers & Harpending, 1992), and goodness‐of‐fit tests of the observed to the estimated mismatch distributions were computed. The possible occurrence of historical demographic expansions was examined by Tajima's *D* (Tajima, 1989) and Fu's *F*s (Fu, 1997) neutrality tests using ARLEQUIN. Fu's *F*s is sensitive to demographic expansion, which usually leads to large negative values (Fu, 1997).

Phylogenetic relationships between geographical samples were estimated by the median‐joining network procedure using the program Network version 4 (http://www.fluxus-engineering.com/). Network analysis effectively portrays the relationships among sequences and allows inference of haplotype genealogies at the population level because they explicitly allow for extant ancestral sequences and alternative connections (Bandelt, Forster, & Rohl, 1999).

Phylogenetic trees to investigate evolutionary relationships were constructed using four methods: neighbor joining (NJ: Saitou & Nei, 1987) using Kimura's two parameter distances (Kimura, 1980), maximum parsimony (MP), maximum likelihood (ML), and Bayesian inference (BI). We used the combined sequences (2,071 bp) without tandem repeats because phylogenetic trees were very similar among separate analyses of cytochrome *b* and control regions (data not shown). European roe deer (*Capreolus capreolus*) was used as the out‐group for phylogenetic tree construction. The NJ, MP, and ML trees were constructed using MEGA 5.05 (Tamura et al., 2011). The MP tree was obtained using Close‐Neighbor‐Interchange with random sequence addition and 10,000 bootstrap replicates.

The most appropriate models of sequence evolution for ML and Bayesian trees were selected with JMODELTEST 2.1.4 (Posada, 2008). The best‐fit model for the ML tree was the general time reversible model (GTR) with Gamma distributed (+G) and proportion of Invariant sites (+I). Consensus ML trees were obtained by Nearest‐Neighbor‐Interchange heuristic searches of 1,000 bootstrap replicates.

Bayesian inference and Bayesian posterior probabilities (BPPs) were estimated using MRBAYES v 3.2.2 (John & Fredrik, 2001). The Hasegawa–Kishino–Yano model (HKY) + G + I was selected as the best‐fit model for the BI tree. Two Markov chains were conducted for 2,000,000 generations, and the tree was sampled every 100 generations with a burn‐in of the first 500 data points. Nodes with bootstrap values (BS) >50% were regarded as sufficiently resolved (Hillis & Bull, 1993). Nodes with BPP >95% were considered statistically significant (Leaché & Reeder, 2002).

Divergence time (*T*) between mtDNA lineages was estimated among clades in the Bayesian tree. *T* was calculated as *K*/(2*r*) (Li, 1997), where sequence divergence (*K*, substitutions/site) was derived from the mean value of *P*‐distance between groups with mean distance determined using Mega 5.2 (Tamura et al., 2011), and *r* is the average mutation rate of mtDNA (12.6 ± 3.2) as proposed by Pesole, Gissi, De Chirico, and Saccone (1999).

## 3 Results

### 3.1 Mitochondrial DNA diversity and genetic divergence

The combined alignment of the mitochondrial control region (923 bp) and cytochrome *b* sequences (1,140 bp) revealed 112 haplotypes, 181 polymorphic sites, and 187 mutations (excluding sites with gaps and missing data). Haplotype distribution and estimates of genetic diversity of each population are presented in Tables 1 and 2.

**Table 2:** Estimates of genetic diversity of regional Siberian roe deer populations

Population	*N*	Combined sequenceCR + Cyt‐*b*			Control region	
		No. Hap.	*h*	π (%)	*h*	π (%)
SKJ	37	9	0.796	0.082	0.251	0.028
SKM	30	19	0.959	0.491	0.915	0.699
RPRA	51	44	0.993	0.769	0.984	0.935
RYA	24	8	0.841	0.974	0.786	1.229
RSMG	22	22	1	0.899	0.991	1.261
RARN	9	5	0.722	0.745	0.722	0.960
RUKO	46	8	0.843	0.884	0.827	0.988
Total	219	112	0.982	0.968	0.961	1.200

Genetic diversity of control region also is presented for comparison with previous studies. See Table 1 for regional abbreviation.

*N*, sample size; No. Hap., Number of haplotypes; *h*, haplotype diversity; π, nucleotide diversity.

Most Central and Eastern Siberian roe deer (RPRA, RSMG, and SKM) did not share haplotypes, except for Sokhondinsky, Russia (RSO), in which one haplotype (Hap98) was shared with Altay, Russia (RAL). On the other hand, the Western Siberia and Ural (RARN and RUKO) populations shared several haplotypes with each other. Yakutia, Russia (RYA) and Jeju Island, Korea (SKJ) were represented by a number of common haplotypes within each population, but haplotypes were not shared among themselves or with other populations (Table 1).

In both combined and control region sequences, the highest levels of genetic diversity, apart from combined nucleotide diversity, were observed in the Trans‐Baikal region (RSMG). Russian Far East (RPRA), Yakutia (RYA), and Ural (RUKO) populations showed moderate to high levels of haplotype diversity (*h *=* *0.841–0.993 for combined sequences and 0.786–0.984 for the control region) and nucleotide diversity (π = 0.769%–0.974% for combined sequences and 0.935%–1.229% for the control region). Mainland Korea (SKM) was characterized by relatively low nucleotide diversity (π = 0.491% and 0.699% for combined sequences), but high haplotype diversity (*h *=* *0.959 and 0.915 for the control region) compared with other populations. Jeju Island, Korea (SKJ), showed the lowest level of genetic diversity for both combined sequences and the control region.

Levels of pairwise population differentiation, *F*_ST_, ranged from 0.037 (RPRA vs. RSMG) to 0.661 (SKJ vs. SKM) (Table 3). Significant pairwise population differentiation was observed between South Korea, Jeju (SKJ), and the other six populations. Other populations that differed significantly from all others were those from the western part of geographical range—Ural (RUKO) and Western Siberia (RARN). However, the genetic differentiation between these two populations was not statistically significant. Pairwise *F*_ST_'s between eastern populations of *C. pygargus* (RSMG, SKM, and RPRA) were not statistically significant, except for SKM versus RSMG. Yakutia, Russia (RYA), was significantly differentiated from most populations (SKM, RARN, and RUKO), but not the Trans‐Baikal region (RSMG). Also, genetic differentiation between Yakutia, Russia (RYA), and the Russian Far East (RPRA) was low (0.109) but significant.

**Table 3:** Pairwise estimates of genetic differentiation between Siberian roe deer populations

	SKJ	SKM	RPRA	RYA	RSMG	RARN	RUKO
SKJ	–	*	*	*	*	*	*
SKM	0.661	–	NS	*	*	*	*
RPRA	0.519	0.054	–	*	NS	*	*
RYA	0.591	0.203	0.109	–	NS	*	*
RSMG	0.588	0.106	0.037	0.040	–	*	*
RARN	0.637	0.413	0.287	0.252	0.232	–	NS
RUKO	0.528	0.382	0.302	0.218	0.261	0.131	–

See Table 1 for regional abbreviation. Population pairwise *F*_ST_ are below the diagonal.

*p‐*Value estimation (above the diagonal) after Bonferroni correction (*, *p *<* *.002; NS: not significant [*p *>* *.05]).

### 3.2 Phylogenetic analysis of mitochondrial haplotype

**Figure 3:**
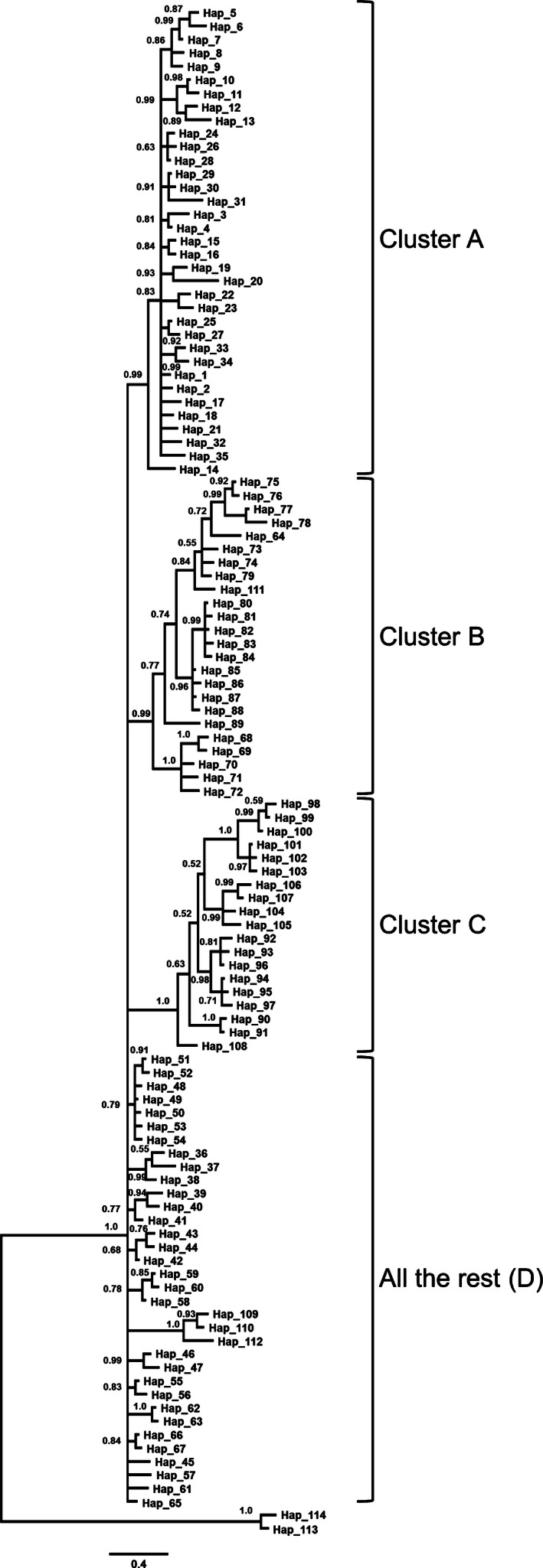
Bayesian (MCMC) haplotype tree of Siberian roe deer based on combined mtDNA control region (923 bp) and cytochrome *b* (1,140 bp) sequences. Bayesian posterior probability is shown for branches with >50% support

Phylogenetic trees using NJ, MP, ML, and Bayesian approaches generated similar patterns of major branches, and therefore, only the Bayesian tree is presented. The Bayesian tree revealed three major haplogroups with strong posterior probability values >.99 and additional 10 minor clusters and singletons (Fig. 3). These minor clusters and singletons mainly originated from eastern populations (SKM, RPRA, and RSMG) that do not differ from one another according to pairwise *F*_ST_'s. Thus, we regarded these clusters as “all the rest” rather than separating small clusters, and they are designated as “group D” in further analyses. Geographical analysis of haplogroup distribution (Fig. 2, Table 4) indicated that none of the clusters, including haplotypes from group D, were limited to a single geographical population. The Jeju Island population consisted only of haplogroup B haplotypes, but this haplogroup was also represented in all populations except mainland South Korea (SKM). Haplogroup A and haplotypes from group D were found mainly in the eastern part of the *C. pygargus* range. Interestingly, a high proportion of haplotypes belonging to haplogroup B was detected in the two populations on the western and eastern periphery of the species’ range—in the Urals (RUKO) and on Jeju Island (SKJ). Haplogroup C was found throughout all regions, except SKJ.

**Table 4:** Distribution of haplotypes in each region among clusters (i.e., haplogroups) revealed by Bayesian tree analysis (Fig. 3)

Haplogroup	No. Hap	Population
A (Red)	35	SKM(5), RPRA(14), RYA(4), RSMG(12)
B (Yellow)	24	SKJ(9), RPRA(4), RYA(1), RSMG(3), RARN(3), RUKO(6)
C (Blue)	19	SKM(2), RPRA(9), RYA(3), RSMG(3), RARN(1), RUKO(2)
All the restD (Gray)	34	SKM(12), RPRA(17), RSMG(4), RARN(1)

See Table 1 for regional abbreviations. In parentheses: number of haplotypes in each geographical population. No. Hap., number of haplotypes.

**Figure 4:**
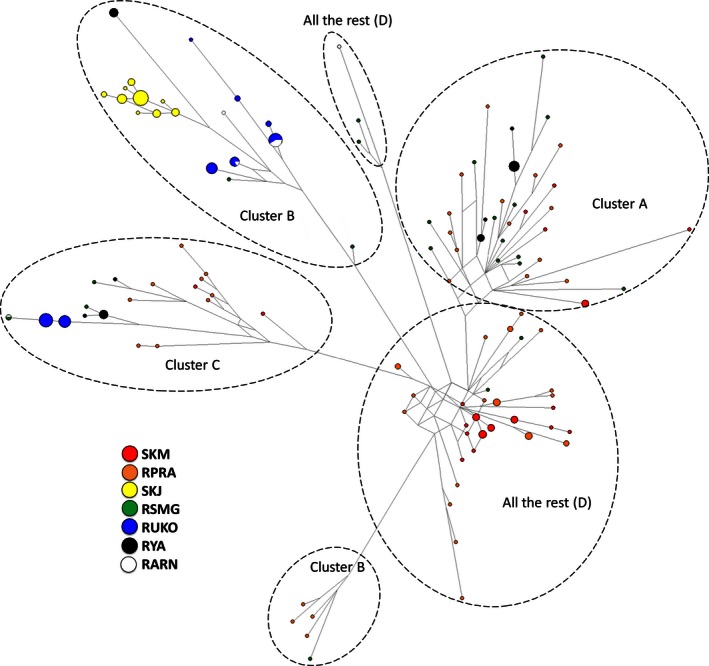
Median‐joining network based on the haplotype data of each individual Siberian roe deer. Branch lengths are scaled to the number of nucleotide substitutions, and size of circles is proportional to haplotype frequency. Dotted lines indicate haplogroup clusters of the phylogenetic tree (Fig. 2)

The median‐joining network was star‐shaped (Fig. 4), with haplotypes from group D connected to all other clusters. Clusters A, B, and C are not interconnected and are connected to group D with long branches, indicating large numbers of missing mutation steps. This relationship is mirrored by divergence times. Clusters A, B, and C have longer divergence times from each other than does each from group D (Table 5). All clusters have similar divergence times from the out‐group, although haplotypes from group D have the shortest.

**Table 5:** Estimates of divergence time among haplogroups derived from Bayesian tree

Divergence point	Estimated sequence divergence (*K* ± *SE*, %)	Estimated time of divergence (*T*, 10^3^ years)
A versus B	1.096 ± 0.160	435
A versus C	1.401 ± 0.216	556
A versus D	0.743 ± 0.122	295
B versus C	1.376 ± 0.210	546
B versus D	0.893 ± 0.130	354
D versus C	1.199 ± 0.194	476
A versus Out	3.941 ± 0.422	1,564
B versus Out	3.820 ± 0.401	1,516
C versus Out	3.971 ± 0.403	1,576
D versus Out	3.585 ± 0.395	1,423

**Figure 5:**
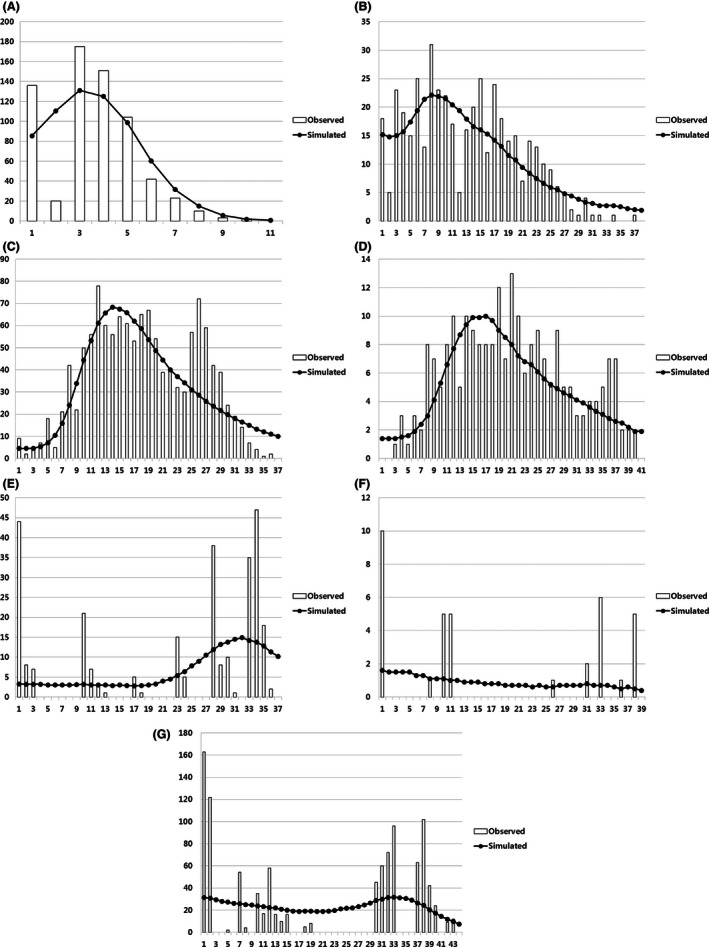
Mismatch distribution of each regional population under the sudden expansion model (A) SKJ; (B) SKM; (C) RPRA; (D) RSMG; (E) RYA; (F) RARN; (G) RUKO. Mismatch distributions based on pairwise site differences between sequences. The expected curve (solid line with dot) was obtained from simulated values computed from data under the model of demographic expansion. See Table 1 for regional abbreviations

Different tests for demographic fluctuations in Siberian roe deer populations showed various aspects of population growth (Table 6, Fig. 5). Analyses of neutrality tests and mismatch distributions show a signature of recent demographic growth for eastern populations (RPRA, RSMG, and SKM) and Jeju Island (SKJ). For these groups, neutrality tests (Tajima *D* and Fu's *Fs*) showed negative values, and Fu's *F*s for RPRA and RSMG differed significantly from that expected under the null hypothesis of selective neutrality. Such results are typical in the case of incomplete lineage sorting or ancestral shared polymorphism under sudden population expansion (Table 6). Although Fu's *Fs* for SKM and SKJ did not differ significantly, the one‐sided bell shape of the mismatch distribution suggests more recent population growth than in other eastern populations (RPRA and RSMG) (Fig. 5).

**Table 6:** Tests for demographic fluctuations of Siberian roe deer populations in each region

Population	*N*	*D* (*p*‐value)	*F*s (*p*‐value)	*r*
SKJ	37	−0.248 (.45)	−0.816 (.39)	.101 (.101)
SKM	30	−1.265 (.08)	−2.143 (.23)	.011 (.751)
RPRA	51	−1.093 (.13)	−20.88 (.00)*	.003 (.548)
RYA	24	1.416 (.95)	10.25 (.99)	.096 (.000)
RSMG	22	−1.130 (.12)	−8.801 (.00)*	.006 (.943)
RARN	9	−1.006 (.15)	4.694 (.97)	.221 (.053)
RUKO	46	2.611 (.99)	18.27 (1.00)	.046 (.006)
Total	219	−0.102 (.41)	0.082 (.50)	–

See Table 1 for regional abbreviations. *D*: Tajima *D*,* Fs*: Fu's *F*s (*p < .05), *r*: raggedness value (*p*‐value in parenthesis) from mismatch analysis.

## 4 Discussion

### 4.1 Genetic diversity and demographic history

In this study, we investigated and compared genetic parameters for populations from most parts of the geographical range of *C. pygargus*. Samples from Western Siberia (RARN) and the Trans‐Baikal region (RSMG) represent populations from the central part of the species’ modern distribution; populations from the Russian Far East (RPRA) and South Korea mainland (SKM) represent the eastern part. Samples from Jeju Island (SKJ) represent an isolated population. The roe deer in Yakutia (RYA) is situated on the northern periphery of the species range. To the west from Ural Moutains Siberian roe deer are sympatric with European roe deer (*Capreolus capreolus*) (Danilkin, 1999; Zvychaynaya, Volokh, Kholodova, & Danilkin, 2013). Thus, the population of Siberian roe deer in Ural region (RUKO) can be treated as situated close to the western periphery of the geographical range of *C. pygargus*.

Relative comparisons of genetic diversity estimates among other species would help in understanding of the present genetic structuring of Siberian roe deer. Only comparisons of genetic diversity we found in the mitochondrial control region with previous genetic studies of Siberian and European roe deer (intraspecific) are possible. Most Siberian roe deer populations in this study revealed similar levels of haplotype and nucleotide diversity, compared to those previously reported for Siberian roe deer (*h *=* *0.93, π = 1.2%; Randi et al., 1998), (*h *=* *0.872, π = 1.1%; Xiao et al., 2007), (*h *=* *0.98, π = 1.66%; Vorobieva et al., 2011), (*h *=* *0.943, π = 1.1%; Lorenzini et al., 2014). Also, similar levels of genetic diversity were observed in other *Cervidae*, such as European roe deer (*h *=* *0.93, π = 1.1%; Randi et al., 1998), (*h *=* *0.971, π = 1.1%; Randi et al., 2004), (*h *=* *0.942, π = 0.95%; Lorenzini et al., 2014), sika deer (*Cervus nippon*) (*h *=* *0.932, π = 1.06%; Wu, Wang, & Fang, 2004), Eld's deer (*C. eldi*) (*h *=* *0.81–0.89, π = 1.4%–2.4%; Balakrishnan, Monfort, Gaur, Singh, & Sorenson, 2003), and reindeer (*Rangifer tarandus*) (*h *=* *0.987, π = 1.8%; Kholodova, Kolpashchikov, Kuznetsova, & Baranova, 2011).

Grant and Bowen (1998) suggested that differences between haplotype and nucleotide diversities can be employed as a means of assessing the demographic history of populations. In both the combined and the control region sequences, the Trans‐Baikal region (RSMG) exhibited the highest levels of genetic diversity in most diversity estimates, except combined nucleotide diversity. This result may be attributable to mixed samples from historically split populations or to stable populations with large long‐term effective population sizes (Grant & Bowen, 1998). High nucleotide diversity of Siberian roe deer at the northern periphery of its geographical range in Yakutia (RYA) may belong to the former scenario as it was reported that the population there is a mixture of two subspecies, *C. p. pygargus* and *C. p. tianschanicus* (Argunov, 2013; Boeskorov & Danilkin, 1998). Co‐occurrence of two subspecies could result in high genetic diversity in a mixed sample.

A moderate level of genetic diversity from western (RUKO) parts of the range may reflect relatively stable populations. This suggestion is supported by the mismatch distribution and neutrality tests. Relatively low genetic diversity in the sample from Western Siberia and Western Altay regions (RARN) could be explained by the small sample size. Siberian roe deer from Jeju Island (SKJ) showed the lowest level of genetic diversity in this study and compared to other species. This is presumably due to its geographical isolation (Jo, Kim, Choi, & Oh, 2012) and a small founder population originally colonizing Jeju Island. The Mainland Korea (SKM) population showed relatively high haplotype diversity and low nucleotide diversity among the populations sampled. This may be attributable to a rapid population expansion after a period of low effective population size caused by a demographic bottleneck (Grant & Bowen, 1998). Support for a population expansion is also indicated by the mismatch distribution, and by the Tajima *D* and Fu's *Fs* values.

### 4.2 Phylogenetic relationships

Our phylogenetic trees and median‐joining network revealed three main trends in genetic relationships between geographical samples of Siberian roe deer. First, we cannot treat any of the haplogroups as ancestral to the others. It is possible the three main haplogroups and additional minor clusters in group D could have formed independently from the out‐group with similar divergence times. Although these minor clusters (D) occupy the center of the star‐like shape in the median‐joining network, analysis of divergence time does not allow treating it as an ancestral. It is more likely that all haplogroups separated from a common ancestor at approximately the same time. The position of the haplogroups in the median‐joining networks suggests that all samples’ haplotypes and haplogroups could have originated from basal haplotypes not present in this study. Minor clusters (D) are probably the closest to this ancestral group, while groups A–C changed significantly since the time of divergence from this putative ancestor.

Second, Siberian roe deer in the area from the Urals to Pacific Ocean did not display unambiguous geographical separation of maternal lineages in the phylogenetic tree and network. This could be explained by incomplete lineage sorting of mtDNA, which would not produce predictable biogeographical patterns (Funk & Omland, 2003; Toews & Brelsford, 2012). On the other hand, geographical distribution of the haplogroups indicated that populations to the east of Lake Baikal have different haplogroup composition than those in Central and Western Siberia (RARN). In addition, among the haplogroups and minor clusters detected in Siberian roe deer, all exist in the central (RSMG) and eastern populations, but only two haplogroups, B and C, are in the Western populations. In particular, eastern populations are mainly represented by haplogroup A and minor clusters (D), which are not present in “Western” populations. This different genetic composition between eastern and Western populations is also supported by the *F*_ST_ value indicating significant genetic differentiation between the two populations. According to Zvychainaya et al. (2011), there are two main haplogroups found in the area from the Urals to Baikal, and these groups probably correspond to haplogroups B and C in our study. The single haplogroup detected from the area of Lake Baikal to the Pacific Ocean in that study may correspond to haplogroup A in our study. Similar to our results, the Lake Baikal region was where “eastern” and “western” haplogroups were found together. However, Zvychainaya et al. (2011) showed that the western part of the species range (the Urals and Western Siberia) harbored a genetically unrelated haplogroup to the Russian Far East and Yakutia. In contrast, our study demonstrated that three haplogroups and group D are found in central and eastern populations, and subsets of these haplogroups (B and C) are found in Western populations. The geographical distribution of haplogroups coincides with the ranges of subspecies *C. p. pygargus* (to the west of Lake Baikal) and *C. p. tianschanicus* (to the east of Lake Baikal), which were previously described based on morphological and cytogenetic traits (Danilkin, 1999; Groves & Grubb, 2011).

The third trend is the genetic composition of the populations in the Urals region (RUKO) and Yakutia (RYA), as well as of the isolated population on Jeju Island (SKJ). Samples from SKJ, RUKO, and RYA, respectively, had one, two, and three haplogroups. Interestingly, the only haplogroup (haplogroup B) detected in Jeju Island was also one of the main haplogroups found in the peripheral populations of the Urals and Yakutia, while it was not observed in the nearby Mainland Korea (SKM) population. This is the first finding that part of the Yakutia (RYA) population is genetically related (shares a haplogroup) to Jeju Island (SKJ). Migration of roe deer to the Jeju Island could have taken place only during periods of glaciation when the island was connected to the continent; thus, haplogroup B may represent one of the oldest genetic lineages. Interestingly, it constitutes the highest proportion of haplogroups mainly in peripheral populations (RUKO and SKJ). A similar pattern of distribution of mtDNA haplotypes was recently reported for another widely distributed mammal species—the European badger, *Meles meles*, where one of the haplogroups was found in the northern and eastern part of the species range, Ireland, but not in nearby Britain (Frantz et al., 2014). Haplogroup A was also found in Yakutia, Russia (RYA), which was a common haplogroup in the eastern and southeastern parts of the species range. Such genetic composition of Yakutia populations could result from several “waves” of species expansion into these territories.

### 4.3 Distribution scenarios of Siberian roe deer

There are several possible scenarios that could explain the observed distribution of genetic lineages of Siberian roe deer. One possible scenario is that all the analyzed samples originated from the same ancestral group, which was preserved in refugia during the periods of climate change in the Pleistocene. Possible locations of the putative ancestral group include the mountains of the southern Siberia, particularly the Altay, Tyan‐Shan, and Sayan mountains. This suggestion is supported by the distribution of several minor clusters (group D), which is present only in the central‐eastern part of the species’ range, and also by the fact that all haplogroups are present only in samples from Trans‐Baikal and Northern Mongolia (RSMG) and the Russian Far East (RPRA). On the other hand, this suggestion contradicts the lack of eastern haplotypes in Central Siberia in the Zvychainaya et al. (2011) study. This could result from the relatively small sample size (total 20 samples from four regions of southern Siberia and Kazakhstan), or perhaps the refugia were outside the sample collection area, probably further south.

Another explanation of the observed diversity of haplogroups in various parts of *C. pygargus*’ geographical range is that several genetic lineages diverged independently from a common ancestor and were isolated from each other during formation of the large open spaces in Central Asia followed by periods of glaciation (Matjushkin, 1982). In this case, high diversity of haplotypes and haplogroups in the Trans‐Baikal region would be the result of secondary colonization by animals from different areas after the periods of climate change. This fits the results of the phylogenetic tree with formation of main haplogroups from independent lineages, and the mismatch analysis showing signs of recent demographic growth in the populations of south Siberia (RSMG) and Amur region (RPRA). Finally, both of the above mentioned scenarios could have taken place—the observed genetic lineages could have originated from a common ancestor inhabiting the mountains of Central Asia. The lineages could have been isolated during periods of increased aridity and glaciation in the Pleistocene followed by recolonization of Central Siberia. More intensive sampling of the regions of southern Siberia and Kazakhstan could reveal roe deer populations with more diverse genetic composition and help identify haplotypes ancestral to those described in this study.

### 4.4 Taxonomic status of Siberian roe deer on Jeju Island

Our results raise questions about the taxonomic status of the Siberian roe deer inhabiting Jeju Island, which was composed of only one haplogroup (cluster B). Genetic distinction (pairwise *F*_ST_ and haplotype distribution) of the Jeju Island population from all other populations does not allow classification of the Jeju roe deer as *C*. *p. tianschanicus* (Koh, Yang, Yoo, & Chun, 2000), nor as a distinct subspecies as suggested by Koh and Randi (2001) and Park et al. (2014). Siberian roe deer from Jeju Island are indeed different from those of mainland Korea (Lee et al., 2015), but they do not appear to represent a distinct phylogenetic clade, sharing the main haplogroup of Western populations. On the other hand, roe deer on Jeju Island are much smaller than those inhabiting the western part of the range. The total body length and height at the shoulder are almost 1.5 times smaller (144 vs. 96 cm and 92 vs. 57.5 cm, respectively) in Jeju roe deer (Danilkin, 1999; Park, Lee, Kim, & Oh, 2011). Genetic similarity associated with obvious morphological differences gives an example of discordance between genetic and morphological evolution in mammals. Lack of correlation between genetic and morphological traits is clearly related to the type of molecular marker, mitochondrial DNA, employed in this study, because comparison of populations based on nuclear markers such as microsatellites revealed clear differences between the Jeju population and roe deer from the western and eastern part of the geographical range (Lee et al., 2015). Therefore, additional and more comprehensive studies will be necessary for clarifying the taxonomic status of roe deer on Jeju Island, Korea.

## 5 Conclusion

Our data show that roe deer in the area from the Urals to the Pacific Ocean are genetically diverse and that the geographical distribution and composition of haplogroups support previously described ranges of the subspecies *C. p. pygargus* and *C. pygargus tianschanicus*. We found that peripheral populations in the northwestern (Urals, RUKO) part of the species range are genetically differentiated from those in the core part of the range in terms of proportional composition of haplotypes. Also, northern (Yakutia, RYA) and northwestern (Urals, RUKO) peripheral populations share the same haplogroup and fall into the same phylogenetic clade with the Jeju Island (SKJ) population. The population of Siberian roe deer on Jeju Island is unique, where conservation of one of the ancient mitochondrial lineages is coupled with specific morphological features.

## Funding Information

Korea Science and Engineering Foundation (Grant/Award Number: 2009‐0080227, NRF‐2008‐314‐C00340).

## Conflict of Interest

None declared.
